# Dual‐Stage Cross‐Flow Filtration: Integrated Capture and Purification of Virus‐Like Particles

**DOI:** 10.1002/bit.28914

**Published:** 2024-12-26

**Authors:** Annabelle Dietrich, Luca Heim, Jürgen Hubbuch

**Affiliations:** ^1^ Institute of Process Engineering in Life Sciences, Section IV: Biomolecular Separation Engineering Karlsruhe Institute of Technology (KIT) Karlsruhe Germany

**Keywords:** continuous processing, cross‐flow filtration, downstream processing, platform process, precipitation, virus‐like particles

## Abstract

Virus‐like particles (VLPs) are a versatile technology for the targeted delivery of genetic material through packaging and potential surface modifications for directed delivery or immunological issues. Although VLP production is relatively simple as they can be recombinantly produced using microorganisms such as *Escherichia coli*, their current downstream processing often relies on individually developed purification strategies. Integrating size‐selective separation techniques may allow standardized platform processing across VLP purification. This study presents an innovative dual‐stage cross‐flow filtration (CFF) set‐up for integrated capture and purification of VLPs, enabling processing solely based on the size‐selective separation techniques precipitation and filtration. The 2 μm/300 kDa MWCO membrane configuration allows the seamless integration of selective VLP precipitation, two consecutive diafiltration steps–first, for washing the VLP precipitates in the first membrane stage, and second, for isolating the re‐dissolved VLPs by continuously removing precipitant and contaminants in the second membrane stage–and ultrafiltration for setting a target VLP concentration. Compared to a single‐stage CFF set‐up, this dual‐stage CFF set‐up with its integrative, automated design demonstrated the capabilities of product accumulation and contaminant handling while maintaining high productivity. Overall, this study represents a significant advancement toward standardized platform processing of protein nanoparticles through precipitation and filtration, and underscores the potential to expand its applicability to diverse biological molecules, unique process conditions, other phase behavior‐dependent processes, and continuous processing.

## Graphical abstract

An innovative dual‐stage cross‐flow filtration (CFF) setup is presented to capture and purify virus‐like particles (VLP). Coupling two CFF units and using a 2 µm/300 kDa MWCO membrane configuration allows for selective VLP precipitation and washing of the precipitate in the first membrane stage combined with subsequent, simultaneous recovery, isolation, and concentration of the re‐dissolved VLP in the second membrane stage. This study spotlights the setup's integrated, automated design and represents a significant step towards standardized platform processing of diverse biopharmaceuticals.

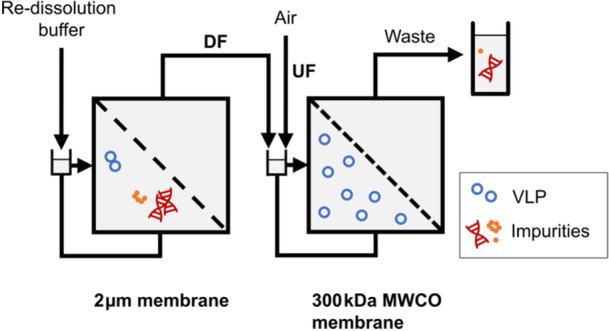

## 1 Introduction

Protein nanoparticles serve as a versatile technology for targeted delivery of various cargos and can also be used as vaccines (Chung, Cai, and Steinmetz 2020; Kim et al. 2023). Similar to the downstream processing of conventional biopharmaceuticals, the downstream processing of protein nanoparticles shares the primary objectives of achieving the required product purity and yield, while effectively managing time and costs in both development and production (Moleirinho et al. 2020). Recent advancements have introduced size‐selective separation techniques such as precipitation and filtration, capitalizing on the notably large size of protein nanoparticles (Hillebrandt and Hubbuch 2023). However, as high as the diversity of protein nanoparticles, so is the challenge of their processing, contributing to the present rarity of standardized platform process development (Moleirinho et al. 2020).

Among protein nanoparticles, virus‐like particles (VLPs) exhibit distinct properties compared to other viral vectors while maintaining their versatility (Qian et al. 2020). VLPs are multimeric structures mimicking the viruses from which they originate (Chackerian 2007; Zeltins 2013) with much lower safety concerns as they lack the viral genome (Nooraei et al. 2021). In addition to their considerable loading capacities, VLPs can undergo surface modifications by genetic engineering or chemical conjugation (Chung, Cai, and Steinmetz 2020). The diversity of VLPs and their expression systems is reflected in the impurity profile of the clarified cell lysate. As a result, the subsequent downstream processing involves a wide array of deployable unit operations for capture, intermediate purification, and polishing steps (Effio and Hubbuch 2015). The main challenge faced by the development of the initial capture step is the targeted removal of process‐related impurities to ensure subsequent separation of their residuals and product‐related impurities with only a small number of purification steps (Effio and Hubbuch 2015). Although steric‐ and size‐exclusion chromatography is employed as a size‐selective separation technique for various VLPs (Hillebrandt and Hubbuch 2023), selective protein precipitation has already established itself as another alternative, particularly for capture. A considerable amount of literature has been published on selective VLP precipitation induced by the addition of ammonium sulfate (AMS) (Tsoka et al. 2000; Koho et al. 2012) or polyethylene glycol (Kim et al. 2010; Zahin et al. 2016; Kazaks et al. 2017) as precipitant. Precipitation itself is highly scalable but typically involves subsequent centrifugation‐based wash and product recovery steps, which suffer from scale‐up challenges and hence are highly insufficient in time and effort. Moreover, precipitate compaction may lead to potential difficulties in re‐dissolution and residual contaminants in the interstitial pellet liquid (Hammerschmidt, Hobiger, and Jungbauer 2016).

Recent developments in the field of precipitation‐based capture processes have led to innovative membrane‐aided approaches using cross‐flow filtration (CFF)‐based diafiltration (DF). Venkiteshwaran et al. (2008) introduced the washing of precipitated immunoglobulins using a cross‐flow microfiltration (MF) membrane to separate them from bovine serum albumin in solution. Similar integrated set‐ups have been reported for concentration and washing of antibody precipitate for batch precipitation (Kuczewski et al. 2011) and sequential precipitation with discontinuous precipitate separation (Hammerschmidt, Hobiger, and Jungbauer 2016). Further developments have been made toward continuous processing, using two‐stage set‐ups with two identical MF membranes connected in series, enabling intensified washing of the antibody precipitate in the second stage (Burgstaller, Jungbauer, and Satzer 2019; Recanati et al. 2023). In all these studies, however, subsequent product recovery was performed separately by either precipitate dilution or centrifugation and pellet re‐dissolution.

A CFF‐based re‐dissolution strategy for integrated product recovery was proposed by Hillebrandt et al. (2020), allowing VLP precipitate to re‐dissolve into VLPs to pass through an MF membrane. At the same time, irreversibly precipitated species are retained. With this set‐up, the original centrifugation‐based procedure is entirely replaced by filtration‐based steps, which is beneficial as filtration offers superior scalability and the capability for disposable manufacturing (van Reis and Zydney 2007; Vicente et al. 2014). Although purity, yield, and productivity improvements have been reported compared to the centrifugation‐based procedure (Hillebrandt et al. 2020), it still faces limitations. In particular, the product‐containing permeate fractions may still have precipitant present, and the ability to integrate additional process steps downstream such as concentration of the re‐dissolved, diluted product is not given. Hence, a subsequent DF for precipitant removal and ultrafiltration (UF) for product concentration might be required. Integrating precipitant removal and product concentration may be achieved by adding a second membrane stage with a smaller MWCO than the first‐stage MF membrane.

Among multistage membrane set‐ups, two‐stage configurations with different membrane MWCOs were specifically employed to isolate the product solely based on size in the second stage while retaining larger species and depleting smaller ones. Cheang and Zydney (2004) presented a set‐up with 100 and 30 kDa MWCO membranes for alpha‐lactalbumin isolation from bovine serum albumin and beta‐lactoglobulin. Similarly, bromelain was isolated from crude pineapple waste mixture using 75 and 10 kDa MWCO membranes (Nor et al. 2016, 2017, 2018). Further, MF was combined with UF to isolate polymerized human hemoglobin from its product‐related high or low‐molecular weight species (LMWS) using an integrated 2 μm/500 kDa MWCO membrane configuration (Cuddington et al. 2022). So far, an integrated two‐stage set‐up for precipitation‐based capture and purification has not been reported.

**Figure 1:**
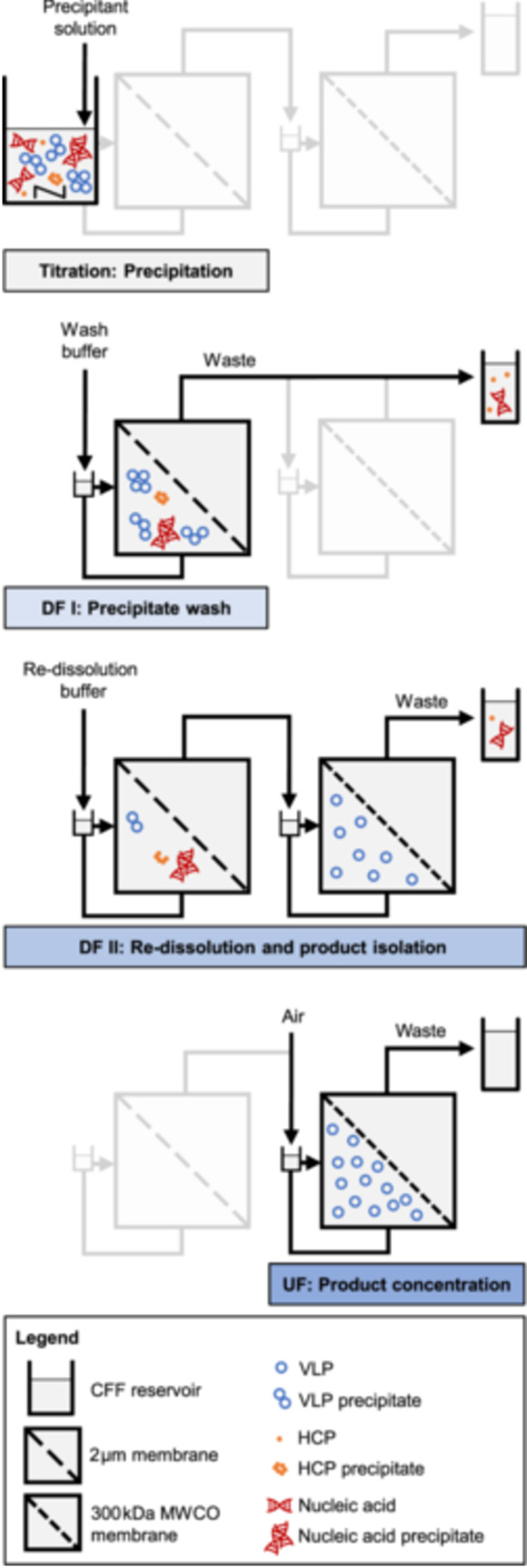
Schematic illustration of the integrated *dsCFF* process. Precipitation by titration in the CFF reservoir is followed by washing of the precipitate by constant‐volume DF using a 2 *μ*m membrane (DFI). Product re‐dissolution by a consecutive DF using the dual‐stage 2 *μ*m/300 kDa MWCO membrane configuration (DFII) allows for integrated product isolation in the second membrane stage. Finally, the setup enables product concentration by subsequent UF. Adapted from Hillebrandt et al. (2020).

In this study, we introduce a dual‐stage CFF (*dsCFF*) set‐up with a 2 μm/300 kDa MWCO membrane configuration for a VLP capture process, enabling the seamless, automated integration of VLP precipitation, precipitate washing, VLP re‐dissolution, as well as their isolation and concentration. A visual illustration of the integrated, consecutive steps is presented in Figure 1. To estimate the impurity profile and the precipitant concentration in CFF‐based processes, we initially provide insights into the VLP re‐dissolution dynamics and the behavior of host‐cell contaminants by a small‐scale, centrifugation‐based screening approach. As a reference process for the VLP construct used in this study, we evaluate a single‐stage CFF (*ssCFF*) process, covering integrated VLP precipitation, washing the precipitate from host‐cell contaminants, and separating re‐dissolved VLPs through a 2 μm membrane from irreversibly precipitated species. To address product concentration and purity limitations during VLP re‐dissolution while maintaining high productivity, we establish the proposed *dsCFF* process by integrating a 300 kDa MWCO membrane as the second membrane stage. Simultaneously with the VLP re‐dissolution and their passage through the first into the second membrane stage, we demonstrate (i) integrated VLP isolation through continuous depletion of precipitant and contaminants, and (ii) integrated VLP concentration by subsequent UF.

## 2 Materials and Methods

### 2.1 Buffers, Solutions, and Virus‐Like Particles

If not otherwise stated, all chemicals were sourced from Merck KGaA (Darmstadt, DE). Buffers and stock solutions were prepared with ultrapure water (PURELAB Ultra, ELGA LabWater, Lane End, High Wycombe, UK), filtered through 0.2 μm pore‐size cellulose acetate filters (VWR International, Radnor, USA) and degassed. The target buffer pH was adjusted with hydrochloric acid. Lysis buffer consisting of 50 mM Tris, 100 mM, 1 mM EDTA (AppliChem GmbH, Darmstadt, DE) at pH 8.0, wash buffer (lysis buffer containing 1.1 M AMS), and re‐dissolution buffer (50 mM Tris, 100 mM NaCl) at pH 7.2 were used for all experiments. High performance liquid chromatography (HPLC) running buffer was 50 mM potassium phosphate buffer at pH 7.4. For polysorbate adjustment and VLP precipitation experiments, 10% (v/v) polysorbate 20 and 4 M AMS stock solutions were used, respectively. For Raman spectroscopy, a set of 12 reference solutions with varying concentrations of AMS (0.1 M increments) was prepared by mixing wash and re‐dissolution buffer at desired levels.

The VLP of interest is composed of C‐terminally truncated wild‐type Hepatitis B core Antigen (HBcAg) proteins, Cp149 (Zlotnick et al. 1996). The plasmid encoding Cp149 was kindly provided by Prof. Adam Zlotnick (Indiana University, USA). A similar procedure of the Cp149 production, capsid harvest, cell lysis, and lysate clarification was conducted as described by Hillebrandt et al. (2020). Clarified lysate solutions containing HBcAg VLPs were thawed, 0.2 μm‐filtered, conditioned, and adjusted to 0.25% (v/v) polysorbate 20. Conditioning comprised absorbance measurement at 280 nm using a NanoDrop 2000c UV/Vis spectrometer (Thermo Fisher Scientific Inc. Waltham, USA) and dilution to a target absorbance with lysis buffer to ensure consistent HBcAg VLP content across centrifugation‐based and CFF‐based experiments.

### 2.2 Centrifugation‐Based Re‐Dissolution Screening

A centrifugation‐based procedure was employed for the re‐dissolution screening, comprising precipitation, washing, re‐dissolution, and recovery steps. In previous capture studies, selective precipitation of the HBcAg VLP studied herein is achieved by the addition of 1.1 M AMS (Valentic, Müller, and Hubbuch 2022; Wegner and Hubbuch 2022; Dietrich et al. 2024). To resolve the VLP re‐dissolution dynamics within the range of 1.1–0 M AMS based on identical experimental conditions, both preceding process steps precipitation and wash were performed individually and parallelized in small‐scale reaction tubes.

Precipitation was conducted by adjusting 500 μL conditioned clarified lysate to 1.1 M AMS. After incubation in an overhead shaker LD‐79 (Labinco, Breda, NL) for 30 min at 10 rpm, precipitates were spun down in a HeraeusPico 17 tabletop centrifuge (Thermo Electron LED GmbH, Osterode am Harz, DE) at 17,000 rcf for 3 min and precipitate supernatants (Prec_S_) were removed. Precipitates were manually re‐suspended in 500 μL wash buffer, incubated at 300 rpm in a thermo‐shaker ThermoMixer Comfort (Eppendorf, Hamburg, DE) for 150 min, and centrifuged using identical settings. The wash supernatants (Wash_S_) were replaced by in total 500 μL, containing a proportional mixture of wash and re‐dissolution buffer, to span the precipitant concentration range from 1.1–0 M AMS in 0.1 M increments intending to mimic buffer exchange during CFF‐based re‐dissolution. Re‐dissolution supernatants (Red_S,*i*_) were recovered after incubation and centrifugation with identical settings. To ascertain full VLP recovery, all pellets of the re‐dissolution conditions were manually re‐suspended in 500 μL re‐dissolution buffer, incubated, and centrifuged for supernatant recovery (Rec_S,0_). Conditioned clarified lysate and all supernatants were analyzed by SDS‐PAGE and HPLC control runs. Analysis by size‐exclusion chromatography (SEC)‐HPLC was exclusively performed for the supernatants of the re‐dissolution and recovery steps.

### 2.3 CFF‐Based Set‐Ups and Processing

**Figure 2:**
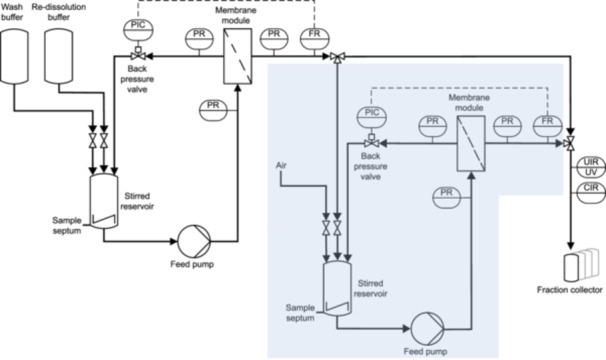
Piping and instrumentation diagram of the CFF set‐up for precipitation‐based capture by constant‐volume DF. In addition to the piping used for consecutive wash and re‐dissolution of the precipitated product (*ssCFF*), a second membrane stage was included before permeate fractionation for the *dsCFF* set‐up, as highlighted in blue. Membrane modules with 2 *μ*m and 300 kDa MWCO were used in the first and second membrane stages, respectively. After re‐dissolution in the *dsCFF* process, the permeate of the first stage was closed to further concentrate the product by UF in the second stage. C, conductivity or control; F, flow rate; I, indicate; P, pressure; R, record; U, multivariate; UV, ultraviolet. Adapted from Hillebrandt et al. (2020).

For the *ssCFF* process, the CFF set‐up relies on the set‐up proposed by Hillebrandt et al. (2020) with minor modifications. The set‐up consisted of a KrosFlo Research KRIIi CFF system with an automatic backpressure valve (Spectrum Labs, Rancho‐Dominguez, US), a 0.2 μm Hydrosart membrane with a filtration area of 200 cm^2^ clamped in the corresponding membrane holder, and a stirred reservoir (all Sartorius Stedim Biotech GmbH, Göttingen, DE). The permeate outlet equipped with a Sensirion Liquid Flow Meter SLS‐1500 (Sensirion AG, Stäfa, CH) and connected to an ÄKTA Start (Cytiva, Uppsala, SE) served as the set‐up for permeate flow control, in‐line ultraviolet (UV) and conductivity measurements, and permeate fractionation. For the *dsCFF* process, a similar, second CFF set‐up was inserted between the one described above and the ÄKTA Start. Here, a 300 kDa MWCO Hydrosart membrane (200 cm^2^, Sartorius Stedim Biotech GmbH) was implemented. Figure 2 displays a detailed piping and instrumentation diagram with the second CFF set‐up highlighted in blue. The permeate flow rate of the first membrane stage was automatically controlled by the backpressure valve controller, using the permeate flow rate data of the flow meter as input along with a custom‐written communication MATLAB 2018b script (The Mathworks, Natick, MA, USA), while the one of the second membrane stage was maintained by manual valve control.

The integrated processes include the steps precipitation, washing of the precipitate by a first DF step (DFI), product re‐dissolution by a second, consecutive DF step (DFII), and–only in the *dsCFF* process–product concentration by UF (cf. Figure 1). Precipitation of the conditioned clarified lysate was conducted by slow, step‐wise pipetting of 4 M AMS stock solution under stirred conditions in the reservoir. At a final concentration of 1.1 M AMS, the solution was incubated for 30 min. All constant‐volume DF steps were conducted with 25 mL diafiltration volume (DV) for six DV, a feed flow rate of 30 mL min^−1^, and a permeate flow rate of 2 mL min^−1^. The first DF step (DFI) for washing the VLP precipitate was performed with wash buffer and the wash permeate was collected as 15 mL fractions at the ÄKTA Start. The second DF (DFII) for VLP re‐dissolution was performed with the re‐dissolution buffer and the permeate fraction size was set to 5 mL or 12.5 mL fractions for the *ssCFF* or *dsCFF* process, respectively. During the *dsCFF* process, retentate samples were taken through an injection plug (Fresenius Kabi, Bad Homburg, DE) every DV. After DF for re‐dissolution in the *dsCFF* process, the first CFF set‐up was disconnected and the remaining retentate in the second membrane stage was concentrated by UF by a factor of 2.5 to a final retentate volume of approximately 10 mL. Samples were taken from the retentates of the 0.2 μm‐membrane after the precipitation, wash, and re‐dissolution, and from the final retentate of the 300 kDa MWCO membrane after UF. After the processes and the removal of the retentates from both CFF systems, both were each flushed with 10 mL re‐dissolution buffer at 50 mL min^−1^ to recover the residual product.

### 2.4 Analytics

Raman spectroscopy provided spectral data to quantify AMS. All Raman measurements were conducted offline using a BioReactor BallProbe (MarqMetrix, Seattle, USA) inserted into the FlowCell Adapter (MarqMetrix) coupled to a HyperFlux PRO Plus 785 with SpectralSoft 3.2.6 (Tornado Spectral Systems, Toronto, CA). The laser power and the exposure time were set to 495 mW and 175 ms, respectively. Spectra were recorded over the spectral range of 200–3300 cm^−1^ with a spectral resolution of 1 cm^−1^ and 50 acquisitions per spectrum. Raman spectra were averaged, normalized at 3299 cm^−1^, baseline‐corrected, and filtered. Baseline correction was performed using a Whittaker filter employing the adaptive smoothness penalized least squares (Zhang et al. 2020) with a *λ* of 6 × 10^7^, a second‐order difference matrix, and 1 × 10^−3^ tolerance. The spectra were smoothed using a Savitzky‐Golay filter (Savitzky and Golay 1964) with a second‐degree polynomial and a window size of 11. A calibration curve created from a set of 12 reference solutions with varying AMS concentrations using the wavenumber 980 cm^−1^ was used to quantify AMS for samples derived from CFF‐based processing.

SEC‐HPLC was used to quantify differently sized species during VLP re‐dissolution/recovery. A Dionex Ultimate 3000 RS UHPLC system (Thermo Fisher Scientific Inc.) was equipped with a BioSEC‐5 column (4.6 × 300 mm, 5 μm, 1000 Å; Agilent, Santa Clara, USA). SEC‐HPLC was performed as previously described by Hillebrandt et al. (2020) with 20 μL injection volume, 0.4 mL min^−1^ flow rate, and 14 min isocratic elution. Samples were either undiluted or diluted 10‐fold and UV spectra were recorded in the wavelength range from 220 to 400 nm. A260/A280 derived from dividing peak areas at 260 nm (A260) by peak areas at 280 nm (A280). Protein concentrations were calculated from A280 using a theoretical Cp149 extinction coefficient of 1.764 gL^−1^ as provided by the ProtParam tool (Gasteiger et al. 2005) and Beer's law.

HPLC control runs without a prefilter and column were performed with 10‐, 20‐ or 40‐fold diluted samples to assess total UV absorbance at 280 nm for all supernatants across all process steps.

Reduced SDS‐PAGE served for quantitative protein analysis and samples were prepared with 50 mM DTT. NuPAGE LDS sample buffer, MES running buffer, and 4%–12% BisTris Mini Protein Gels were sourced from Invitrogen (Carlsbad, USA) and separation was performed on an XCell SureLock Mini‐Cell from the same vendor. Protein bands on the gels were selectively stained using InstantBlue protein stain (US Biological, Salem, USA).

## 3 Results

### 3.1 Re‐Dissolution Screening Reveals Fast Re‐Dissolution of VLPs and LMWS

Following VLP precipitation and precipitate wash at 1.1 M AMS, VLP re‐dissolution was studied over the precipitant concentration range of 1.1–0 M AMS in a small‐scale, centrifugation‐based screening to mimic the buffer exchange during CFF‐based processing.

**Figure 3:**
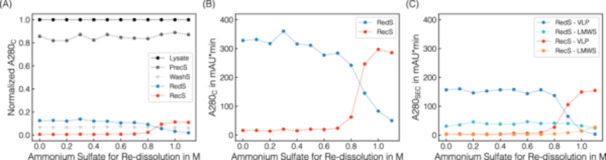
Centrifugation‐based re‐dissolution screening with AMS concentrations ranging from 1.1 to 0 M. Absorbance at 280 nm assessed by HPLC control runs A280_C_ (A), (B) and derived from SEC‐HPLC A280_SEC_ (C) are shown as symbols with their respective assignment to the process step supernatants after centrifugation: precipitation at 1.1 M AMS (Prec_S_), wash at 1.1 M AMS (Wash_S_), re‐dissolution under varied AMS concentrations ranging from 1.1 to 0 M (Red_S_), and recovery at 0 M AMS (Rec_S_). Dashed lines are illustrated only for visual purposes. Normalized A280_C_ values (A) enable estimations of the distribution of all species originally present in the lysate across the individual process steps. For the re‐dissolution and recovery step, absolute A280_C_ values are presented in (B) and A280_SEC_ values shown in (C) represent the individual proportions of VLP and LMWS.

Total UV absorbance values at 280 nm (A280_C_) derived from HPLC control runs are shown upon normalization in Figure 3A, starting from clarified lysate through the supernatants from the precipitation, wash, re‐dissolution, and recovery step. Slightly varying A280_C_ but within constant ranges were observed for both precipitation (~85%) and wash (~6%) supernatants, as expected given the identical procedures and indicating soluble contaminants. The corresponding SDS‐PAGE scans confirm that both supernatants mostly contain host‐cell proteins since diverse protein bands are visible but without the protein band attributed to Cp149 (cf. Supporting Information S1: Figure S1). Additionally, mean A260/A280 ratios of 1.83 for the precipitation and 1.86 for the wash supernatant indicate the presence of host‐cell nucleic acids in both supernatants.

For visual purposes, the observed re‐dissolution dynamics during the re‐dissolution and recovery steps are further resolved and illustrated as absolute A280_C_ in Figure 3B. A dynamic re‐dissolution behavior was observed within the range of 1.1–0.7 M AMS. Absorbance values from the recovery step, performed with solely AMS‐free re‐dissolution buffer, reflect the exact reverse trend, indicating effective VLP re‐dissolution. Averaging A280_C_ from both re‐dissolution and recovery steps across the conditions, ~13% species were re‐dissolved (cf. Figure 3A). It has to be noted that residual pellets were visible in the reaction tubes upon final recovery accompanied by a discrepancy in A260_C_ content, suggesting irreversibly precipitated host‐cell nucleic acids (data not shown).

The re‐dissolved species upon re‐dissolution and recovery were separated and assessed by SEC‐HPLC and the A280_SEC_ courses are illustrated in Figure 3C. For A280_SEC,VLP_, a similar trend as for the A280_C_ (cf. Figure 3B) is observed reflecting the actual VLP re‐dissolution behavior with 0.7–1.1 M AMS as dynamic range. However, VLP re‐dissolution is accompanied by re‐dissolution of other LMWS. These findings correspond with those from SDS‐PAGE (cf. Supporting Information S1: Figure S1). Interestingly, approximately 50% of these LMWS already re‐dissolve at 1.1 M AMS, which is representative of a second wash step and in turn resulted in the highest VLP purity upon recovery for the 1.1 M AMS condition. Furthermore, A260/A280 values below 0.65 indicate that the presence of proteins predominates over that of nucleic acids upon re‐dissolution and recovery. Overall, a mean VLP recovery of 1.25 ± 0.05 mg per 500 μL conditioned clarified lysate was observed considering the A280_SEC,VLP_ apart from the dynamic range.

Lastly, discrepancies were observed between A280_C_ and the sum of A280_SEC,*i*_ (cf. Figure 3B,C) in terms of absolute absorbance values, suggesting the presence of species being even larger than VLPs and thus being retained in the SEC pre‐column filter. The presumption that host‐cell nucleic acids were present in the re‐dissolution and recovery supernatants is underlined by the observed A260/A280 ratios ranging from 0.8 to 1.0 in the HPLC control runs and by their interferences at 280 nm which might contribute to the observed A280_C_.

In summary, the re‐dissolution screening provides insights into the VLP re‐dissolution dynamics and the content of simultaneously present LMWS, while also indicating irreversibly precipitated host‐cell nucleic acids. The re‐dissolution of VLPs in the upper precipitant range suggests fast re‐dissolution during CFF‐based processing, accompanied by significant residual precipitant content.

### 3.2 Dual‐Stage CFF Isolates and Concentrates VLP

**Figure 4:**
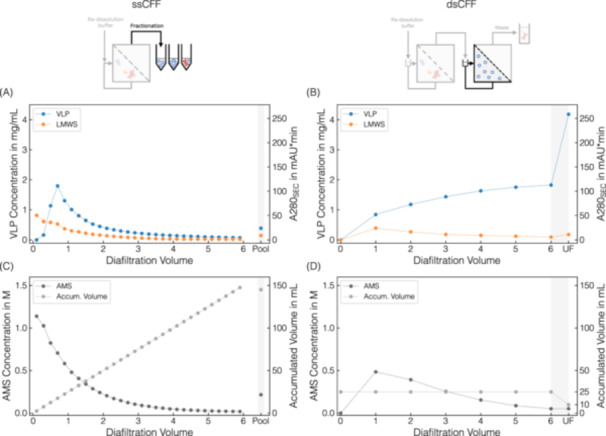
Comparison of *ssCFF* and *dsCFF* processes. SEC‐derived VLP concentration and 280 nm absorbance of LMWS are displayed against DV for the *ssCFF* process in (A) and *dsCFF* process in (B). In (C), (D), the corresponding Raman‐derived AMS concentration and the accumulated product volume are shown. For visual purposes, the data points are connected with dashed lines. Data points from the *ssCFF* process (A, C) derived from product‐containing permeate fractionations in 5 mL scale. The final data by pooling all VLP‐containing fractions are shaded with gray bars (Pool). Data points from the *dsCFF* process (B, D) were obtained by sampling at each DV of the product‐containing retentate in the second membrane stage. The *dsCFF* set‐up allowed for integrated UF of this second‐stage retentate, highlighted with gray bars (UF).

The VLP precipitation and the CFF‐based wash by DF were reproducibly performed and allowed for efficient contaminant removal as further presented in Supporting Information S1 Section 1.1, providing a consistent basis for the subsequent, second DF for VLP re‐dissolution. The courses of VLP re‐dissolution for both processes–the reference *ssCFF* and the novel introduced *dsCFF* process–are illustrated in Figure 4, with the resulting performance indicators summarized in Table 1.

**Table 1:** Overview of final *ssCFF* and *dsCFF* process data. For the *dsCFF* process, data were evaluated at different stages of the process: after DF for six DV (DF), after UF (DF/UF), and after a flushing procedure of the second membrane stage (DF/UF*). VLP concentration, purity, and relative VLP recovery derived from SEC. The relative VLP recovery is relative to the *ssCFF* process. The A260/A280 was determined by HPLC control runs. Raman‐derived spectral data were used for AMS quantification. For each column, values representing the best conditions are highlighted in bold.

	VLP concentration	Purity	A260/A280	AMS concentration	Relative VLP recovery
	g L^−1^	%	—	M	%
*ssCFF*_Pool_	0.39	73.0	0.81	0.22	100
*dsCFF*_DF_	1.82	94.5	0.73	0.05	81
*dsCFF*_DF∕UF_	4.18	**95.3**	**0.72**	0.05	74
*dsCFF*_DF∕UF*_	**5.04**	94.8	0.73	**0.04**	**90**

For the *ssCFF* process, the buffer exchange into re‐dissolution buffer enables the species to re‐dissolve and pass through the 2 μm MF membrane for permeate fractionation while simultaneously, aggregated or irreversibly precipitated species are retained. In general, the VLP concentration in the permeate fractions increases sharply at the beginning and then decreases steadily from approximately 0.75 DV onwards throughout the entire process, whereas the proportion of LMWS continuously decreases (cf. Figure 4A). For LMWS, a mean A260/A280 of 1.15 indicates a considerable amount of both host‐cell proteins and nucleic acids. Raman spectral analysis revealed the decrease in AMS through buffer exchange following the typical constant‐volume DF principle (cf. Figure 4C). The in‐line recorded UV and conductivity data from the permeate depicted in Supporting Information S1: Figure S2B qualitatively exhibit the same trends as the off‐line SEC and Raman data derived from the permeate fractions, respectively. As expected from the re‐dissolution screening, the VLPs re‐dissolve fastly (cf. Section 3.1), and the highest VLP concentrations were found in the permeate fractions 3 to 5 corresponding to 0.5–1 DV. However, these permeate fractions are accompanied by LMWS being present as well as relatively high AMS concentrations of 0.6–0.8 M AMS. Alternative pooling strategies consistently trade off one characteristic for another. The final data, achieved by pooling all VLP‐containing permeate fractions, are highlighted by gray bars (cf. Figure 4A,C) and listed in Table 1 (*ssCFF*). The accumulated permeate volume of 145 mL with a VLP concentration of 0.39 g L^−1^ still exhibits 0.22 M AMS and LMWS contaminants, leading to a purity of 73 %. It has to be noted that the purity might have been increased by extending the wash step beforehand. However, the delayed decline in 280 nm absorbance of LMWS between 0.5 and 0.7 DV (cf. Figure 4A) still indicates prior precipitated and re‐dissolved LMWS shortly before or simultaneously to VLPs. This finding aligns once again with the outcome of the re‐dissolution screening, where a similar LMWS re‐dissolution behavior was found (cf. Section 3.1). Overall and despite the limitations in product dilution, product purity, AMS depletion, and alternative pooling strategies, the *ssCFF* process is applicable for VLP constructs requiring a considerably high amount of AMS for precipitation.

The *ssCFF* set‐up serves as the foundation for the *dsCFF* set‐up (cf. Figure 1), where an additional 300 kDa MWCO membrane stage is introduced between permeate outlet and fractionation (cf. Figure 2). Next to the ability to separate re‐dissolved species from aggregated or irreversibly precipitated species in the first stage, the set‐up enables the integrated isolation of VLPs in the second stage retentate by depleting precipitant and contaminants. The VLP concentration increases continuously through DF with the greatest increase within the first two DV, while the LMWS concentration starts to decrease again after the first DV (cf. Figure 4B). The depletion of the precipitant AMS follows the same pattern as the depletion of LMWS (cf. Figure 4B,D). LMWS and AMS passed through the second stage membrane, which was further supported by SEC/SDS‐PAGE and Raman analysis of the permeate fractions (data not shown). To enable this integrated process step, the second membrane stage was rinsed with 1 DV (25 mL) of re‐dissolution buffer beforehand, and through constant‐volume DF, the total volume remained at 25 mL (cf. Figure 4D). After six DV (cf. Table 1, *dsCFF*_DF_), a purity of 94.5% was achieved and AMS could be reduced to 0.05 M. It has to be noted that both purity and AMS depletion could have been further improved by further extending this DF step. The progression of integrated UF of the second stage retentate (cf. Table 1, *dsCFF*_DF/UF_) is highlighted with gray‐shaded bars for visual clarity in Figure 4B,D. A VLP concentration by a factor of 2.3 was observed through the 2.5‐fold reduction of the retentate volume, while AMS was further depleted. Interestingly, and contrary to the assumption that the depletion of LMWS might also continue almost unhindered, the LMWS concentration significantly increased during UF. As a result, only a marginal improvement of less than 1% in purity was observed. Given the relatively small process volume compared to the CFF system and membrane area, a flush with 10 mL of re‐dissolution buffer was performed to maximize the recovery of remaining deposits on the retentate side of the second membrane stage. Theoretical values are summarized in Table 1, *dsCFF*_DF∕UF*_. A VLP concentration of 4.5 g L^−1^ is accompanied by slightly lower purity and residual AMS, whereas the recovered VLP significantly increased to 90%. Despite the VLP recovery being increased by the flushing procedure, it is still lower than in the *ssCFF* process.

The reproducibility of the re‐dissolution processes (cf. Supporting Information S1 Section 1.2) allows for a comparison of both. Compared to the *ssCFF* process, the *dsCFF* process achieved a reduction of residual AMS by a factor of 4, a depletion of LMWS by a factor of 8, and a 13‐fold VLP concentration. In summary, the *dsCFF* set‐up using a 2 μm/300 kDa MWCO membrane configuration successfully integrated VLP isolation and concentration simultaneously to the CFF‐based re‐dissolution, which was not given by the *ssCFF* set‐up.

## 4 Discussion

### 4.1 Screening of Solubility‐Driven Processes

In solubility‐driven processes, it is crucial to study the product behavior and the behavior of its contaminants under all process conditions. Although precipitation or crystallization are frequently screened processes for various biological molecules (Baumgartner et al. 2015; Huettmann et al. 2015; Barros Groß and Kind 2018; Großhans, Suhm, and Hubbuch 2019; Gu et al. 2020; Wegner, Zimmermann, and Hubbuch 2022), their re‐dissolution behavior has been rarely studied. In the scope of transferring the centrifugation‐based VLP precipitation/wash/re‐dissolution process to a CFF‐based process for buffer exchange, the VLP re‐dissolution screening was designed to unveil the re‐dissolution dynamics across a range of precipitant concentrations. Studies with a similar screening objective, mimicking a process by resolving the additive concentrations, have been reported for fed‐batch VLP precipitation (Hillebrandt et al. 2020; Dietrich et al. 2024) or evaporative protein crystallization (Barros Groß and Kind 2018), among others. Such screenings allow for estimations of the applicability and limitations of the actual process.

For VLP precipitation, quasi‐instantaneous changes upon setting the precipitant condition were observed, which is consistent with observations from a chimeric HBcAg VLP (Hillebrandt et al. 2020) or other truncated wild‐type HBcAg VLPs with variations of the nucleic acid binding regions (Valentic, Müller, and Hubbuch 2022). Comparing the VLP precipitation behavior (Dietrich et al. 2024) with the here screened re‐dissolution behavior, the dynamic range is within the same precipitant concentrations, hence the respective sigmoidal curves exhibit inverse behavior. This VLP re‐dissolution in the higher precipitant range indicates early re‐dissolution during CFF‐based buffer exchange, along with a substantial amount of residual precipitant. Further, slight co‐precipitation and co‐re‐dissolution of LMWS reduce the selectivity of VLP precipitation, thereby diminishing the purity of the recovered product.

Especially in solubility‐driven processes of biological molecules, aggregated or irreversibly precipitated species may form which cannot be recovered. As in our case, indications for irretrievable host‐cell nucleic acids emphasize the need for employing separation techniques such as centrifugation, which is considered the standard procedure, or filtration (Hillebrandt et al. 2020). Overall and in the scope to resolve phase behavior, it is necessary to set the screening conditions individually for fast reactions such as VLP precipitation, or for slower, rather uncontrollable reactions over time such as VLP re‐dissolution after precipitate compaction.

### 4.2 Integrated Dual‐Stage CFF for VLP Re‐Dissolution, Isolation, and Concentration

Applying CFF‐based processing, selective VLP precipitation is followed by a first DF step for washing the VLP precipitates. The methodology of washing the precipitate using an MF membrane originates from Venkiteshwaran et al. (2008), with further innovative contributions enabling product concentration (Kuczewski et al. 2011), sequential processing (Hammerschmidt, Hobiger, and Jungbauer 2016), or intensified washing using serial MF membranes (Burgstaller, Jungbauer, and Satzer 2019; Recanati et al. 2023). Using constant‐volume DF, the wash buffer requires the same precipitant concentration as used for precipitation to ensure that the precipitate remains in its precipitated form. In the scope of selective precipitation, the species that remain soluble are considered contaminants to be removed by depletion through the membrane. Generally, a sufficiently long wash step is expected to result in higher purities than centrifugation‐based processing, as interstitial pellet liquid is circumvented (Hammerschmidt, Hobiger, and Jungbauer 2016). To further investigate residual contaminant handling during the second DF step comparing the *ssCFF* and *dsCFF* set‐ups, the wash was conducted for a fixed number of DV instead of continuing until the in‐line UV signal in the permeate fell below a desired, small enough arbitrary unit as done by Hillebrandt et al. (2020). As a result, a tiny portion of contaminants remained in the retentate. Although Hillebrandt et al. (2020) performed an adequate wash step, they also observed insufficient removal of host‐cell nucleic acids.

The benefits of employing an MF membrane for integrated, CFF‐based precipitate wash with a subsequent, second DF step for VLP re‐dissolution have been thoroughly examined by Hillebrandt et al. (2020). Buffer exchange facilitates the re‐dissolution of VLP precipitates, allowing the VLPs to pass through the MF membrane. In short, preventing pellet compaction and interstitial pellet liquid leads to faster re‐dissolution (Hammerschmidt, Hobiger, and Jungbauer 2016; Hillebrandt et al. 2020), thereby increasing relative productivity. However, the re‐dissolution process by DF results in re‐dissolved VLPs present in a highly diluted form accompanied by residual precipitant in the permeate fractions owing to the previous wash step. Further, co‐re‐dissolved LMWS can not be handled using the *ssCFF* set‐up. Overall, the application of the *ssCFF* set‐up requires highly selective precipitation of the target product, while the dilution through DF and the presence of residual precipitant and LMWS remain insufficiently addressed. Consequently, we confirmed the predictions provided by the re‐dissolution screening on a small scale about the applicability of the process for our VLP construct. For other biological molecules, such screenings may help verify the conditions for applicability.

Applying the *ssCFF* set‐up would require a pooling strategy and sequential processing steps, only achievable through manual intervention. These steps would involve an intermediate UF for volume reduction, DF for residual precipitant and LMWS depletion, and final UF for setting a target product concentration, resulting in a significant drop in productivity. Further and especially in larger scales, this additional intermediate UF step subjects the product to a prolonged processing time. The integrated *dsCFF* set‐up using a 2 μm/300 kDa MWCO membrane configuration allows constant‐volume buffer exchange by DF followed by product concentration through subsequent UF, hence skipping the intermediate UF step and maintaining high productivity. Similar strategies isolating the target product have been reported using two UF membrane stages (Cheang and Zydney 2004; Nor et al. 2016, 2017, 2018), while Cuddington et al. (2022) also used an integrated MF/UF membrane configuration. However, these processes describe simple isolation based on size and do not involve changes in phase behavior.

During the *dsCFF* process, the VLPs are isolated and concentrated in the second stage retentate, while the precipitant and other LMWS are depleted. Moreover, the product volume stays constant, and further concentration of the product by subsequent UF enables the setting of a target product concentration. The depletion of contaminants significantly broadens the applicability of the process, making it applicable for biological molecules where precipitation is not highly selective and contaminants co‐re‐dissolve. Further, effectively addressing precipitant depletion may allow for potential recycling, marking a major advancement toward sustainability.

Throughout UF, a constant concentration of the depleting species in the retentate would have been expected for unhindered depletion. In contrast to the nearly unhindered depletion of the small solute AMS, the LMWS content increased through UF, resulting from a slower depletion than concentration process, which may be attributed to the electrostatic exclusion of proteins (van Reis and Zydney 2007). However, the solute‐specific selectivity of pressure‐driven membrane processes might also be diminished by high concentrations of solutes. Next to concentration polarization describing restricted solvent flux mainly through protein concentration near the membrane surface, proteins also tend to deposit on membrane surfaces, as these are susceptible to fouling (van Reis and Zydney 2007). The membrane surface in the *dsCFF* process is doubled compared to the *ssCFF* process, and hence the surface for deposition as well. The authors suggest that lower VLP recoveries for all evaluated *dsCFF* steps than for the *ssCFF* process are attributable to fouling and system deposits. After DF/UF, the best results in the purity, A260/A280, and residual AMS content were achieved, while a drop in relative VLP recovery was observed. This drop in relative VLP recovery may partly be attributed to VLP deposition on the second‐stage membrane surface through concentration, as higher concentrated products tend even more to fouling (van Reis and Zydney 2007). Flushing the membrane and system with fresh buffer recovers soluble species from the membrane surface, tubing, and reservoir. A relative VLP recovery of 90% could be achieved with higher VLP concentrations and only minor compromises in purity. Product recovery can generally be optimized through reductions of relative membrane accumulations by processing larger volumes or using optimized surface areas.

In the scope of providing a proof of concept for integrated, automated processing applying the innovative *dsCFF* set‐up and to illustrate its versatility, the process was not optimized in terms of transmembrane flux to enhance productivity, membrane surface to product volume/concentration to improve relative VLP recovery, or DV to increase purity. The here performed CFF processes were designed to allow for permeate fractionation by controlling the permeate flow rate to 2 mL min^−1^, enabling full characterization of the process. The transmembrane pressures ranged between 0.01 and 0.03 bar in all processes with feed pressures up to 0.3 bar. Such relatively high backpressure on the permeate side is unusual in CFF processes but attributable to the used permeate capillaries small in diameter, necessary to combine a traditional CFF unit with an ÄKTA system for recording in‐line signals and enabling permeate fractionation. When transferring the here presented *dsCFF* process to larger scales, an abandonment of the set‐up for permeate fractionation allows for further optimizations in the process operating parameters transmembrane flux and membrane surface area.

### 4.3 Versatility of Dual‐Stage CFF

The innovative, integrated *dsCFF* set‐up using an MF/UF membrane configuration may serve as a valuable platform for diverse advancements.

First, it may be applied for precipitation‐based capture and purification processes of other biological molecules such as monoclonal antibodies or viral vectors under the premise that the second membrane MWCO is adjusted to retain the target product selectively. The simultaneous depletion of precipitant and contaminants allows the set‐up application even for (i) biological molecules requiring high precipitant concentrations for precipitation and (ii) processes with contaminant co‐precipitation/‐re‐dissolution. Further, these processes could be even more optimized for productivity by shortening the wash step beforehand.

Second, the integrative design of a *dsCFF* set‐up may be transferable to other phase behavior‐dependent processes than precipitation such as dis‐ and reassembly of VLPs. Lining up several membrane configurations, VLPs could be captured, purified, disassembled, reassembled, and formulated solely CFF‐based and fully integrated.

Third, developing integrated, robust platform processes is one of three key requirements for transitioning toward continuous bioprocessing of VLPs, alongside developing customized unit operations and real‐time monitoring tools (Mittal et al. 2022). The importance of process integration for continuous processing of biopharmaceuticals is further emphasized by Gerstweiler, Bi, and Middelberg (2021), providing a detailed overview of chromatographic and several non‐chromatographic approaches, including precipitation and filtration. Building upon the studies by Burgstaller, Jungbauer, and Satzer (2019) and Recanati et al. (2023), which have already demonstrated continuous precipitation and washing of precipitates for monoclonal antibodies, the integrated *dsCFF* set‐up proposed here may serve as a valuable process extension for further continuous precipitate processing in terms of the product re‐dissolution, isolation, and concentration. Based on precipitation and filtration technologies, implementing such a continuous VLP capture and purification process could offer a beneficial alternative to fully chromatography‐based purification processes of VLPs, such as the fully integrated, continuous capsid protein purification by flow‐through anion‐exchange and periodic counter‐current b&e multimodal chromatography of the modular murine polyomavirus major capsid (Gerstweiler et al. 2022).

In summary, this study significantly contributes toward standardized platform processing through solely size‐selective separation techniques precipitation and filtration, thereby broadening the concept's applicability to diverse biological molecules, extraordinary process conditions, other phase behavior‐dependent processes, and continuous processing.

## 5 Conclusion and Outlook

In conclusion, we present an innovative *dsCFF* set‐up for integrated VLP capture and purification using a 2 μm/300 kDa MWCO membrane configuration. Through CFF‐based processing, selective VLP precipitation is followed by a first DF step for washing the VLP precipitates and a second DF step for VLP re‐dissolution. Here, a fast re‐dissolution of the VLPs accompanied by a high residual precipitant load and co‐re‐dissolved contaminants was suggested by a small‐scale, centrifugation‐based screening. Due to the nature of DF, the reference *ssCFF* process further led to re‐dissolved VLPs present in a highly diluted form in the permeate. Both observed limitations–product purity and concentration–are addressed by the *dsCFF* set‐up with an MF/UF membrane configuration. Along with VLP re‐dissolution and their passage through the MF membrane, the integrated second membrane stage successfully enabled VLP isolation by simultaneous removal of contaminants and precipitant, as well as VLP concentration by subsequent UF. Hence, the *dsCFF* set‐up integrates further polishing of the product pool into one unit operation while maintaining higher productivity compared to sequential unit operations. In comparison to the *ssCFF*‐derived product pool, the proposed *dsCFF* set‐up facilitates 13‐fold product concentration, the reduction of residual precipitant by a factor of 4, and the depletion of contaminants by a factor of 8. The *dsCFF* setup's ability to deplete the precipitant could enable precipitant recycling, marking an additional advancement toward enhanced sustainability. Overall, the developed integrated *dsCFF* set‐up provides a foundation for standardized platform processing exclusively based on size‐selective separation techniques and bears the potential to be applied to diverse biological molecules, extraordinary process conditions, other phase behavior‐dependent processes, and even continuous processing.

## Author Contributions

**Annabelle Dietrich:** conceptualization, methodology, investigation, software, formal analysis, visualization, writing–original draft, writing–review and editing. **Luca Heim:** investigation, software, formal analysis, writing–review and editing. **Jürgen Hubbuch:** conceptualization, supervision, funding acquisition, writing–review and editing.

## Conflicts of Interest

The authors declare that the research was conducted in the absence of any commercial or financial relationships that could be construed as a potential conflict of interest.

## Supporting information

Supporting Information

## Data Availability

The raw data supporting the conclusions of this article will be made available by the authors, without undue reservation.
