# Supplementary material for: Dual‐Stage Cross‐Flow Filtration: Integrated Capture and Purification of Virus‐Like Particles
Source: Biotechnol Bioeng. 2024 Dec 26;122(4):884–94. doi: 10.1002/bit.28914 (PMC11895408; doi:10.1002/bit.28914)
Supplement: Supplementary file 1 — Supporting Information [file BIT-122-884-s001.pdf]

Supporting Information S1

1 SUPPLEMENTARY DATA

1.1 CFF-Based Wash Reproducibility

To recover precipitated virus-like particles (VLPs) after selective precipitation, the applicability of a cross-flow filtration (CFF)-based wash of the precipitate by a first diafiltration (DF) step (DFI) has been assessed. The CFF-based wash aims for the integrated removal of dissolved species while the precipitate is still retained by the 2 µm microfiltration (MF) membrane. In-line permeate data from a representative wash step are shown in Supporting Information S1: Figure S2 (A), since reproducible data were achieved across all conducted processes. In-line conductivity measurements showed a consistent conductivity value of approx. 156 mS cm^{-1} throughout the wash step, indicating the presence of ammonium sulfate (AMS). Raman spectral analysis of the fractionated permeate enabled AMS quantification and underlined the constant AMS concentration of approx. 1.1 M (data not shown), as expected due to a 1.1 M AMS-containing wash buffer. Contaminant removal was monitored by in-line ultraviolet (UV) of the permeate (cf. Supporting Information S1: Figure S2 (A)) and after approx. six diafiltration volume (DV), the signal dropped below 60 mAU. Contaminant removal was further supported by SDS-PAGE of the permeate fractions (data not shown). Overall, VLP precipitation and the CFF-based wash could be reproducibly performed, allowing for efficient contaminant removal and providing a consistent basis for subsequent CFF-based re-dissolution processes.

1.2 CFF-Based Re-Dissolution Reproducibility

Considering the first membrane stage, residual turbidity of the retentate was visible after CFF-based re-dissolution, the second DF step (DFII), suggesting aggregated or irreversibly precipitated host-cell species. Mass balances derived from high performance liquid chromatography (HPLC) control runs of the retentate supernatants revealed $A260_C$ discrepancies, hence underlining this assumption (data not shown), which is in good agreement with findings from the re-dissolution screening. Analysing the retentate supernatants for soluble species, size-exclusion chromatography (SEC)-HPLC analysis revealed VLP concentrations between 0.23–0.26 g L^{-1}, similar A260/A280 ratios of 0.81, and purities between 88–89%, indicating reproducible process operations. However, the authors believe that extending the DF process for re-dissolution could have led to a greater proportion of VLPs passing through the MF membrane, thereby increasing overall VLP recoveries in both processes.

2 SUPPLEMENTARY FIGURES

2.1 Figures

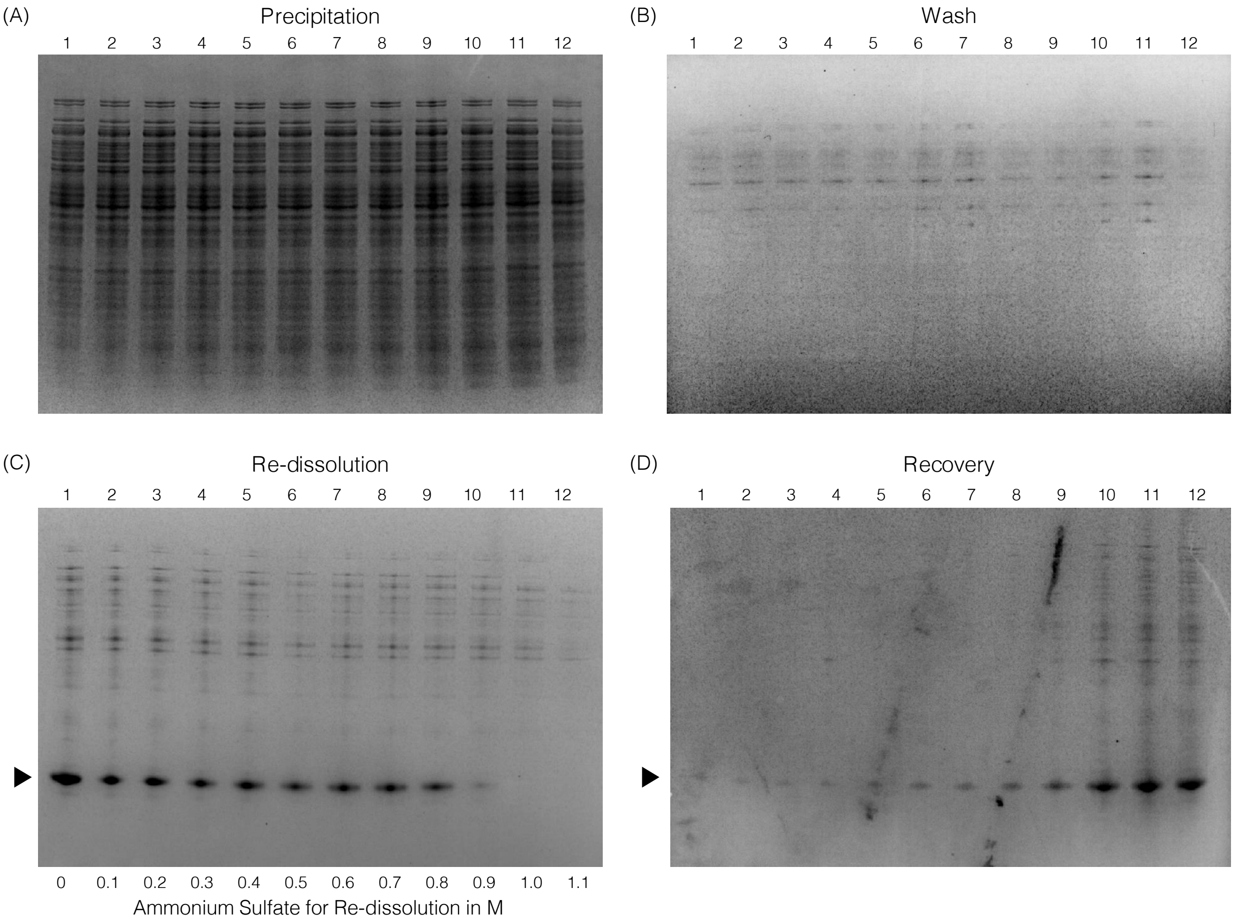

Figure S1. SDS-PAGE gel scans of the VLP re-dissolution screening. Supernatants after the steps precipitation (A), wash (B), re-dissolution (C), and recovery (d) are depicted. The lanes 1-12 correspond to the solutions, for which the AMS concentrations were varied during VLP re-dissolution (C). Note that 1.1 M AMS was consistently deployed for precipitation (A) and wash (B), while VLPs were finally recovered using a buffer without AMS (D). The protein band attributed to Cp149 is indicated by arrows.

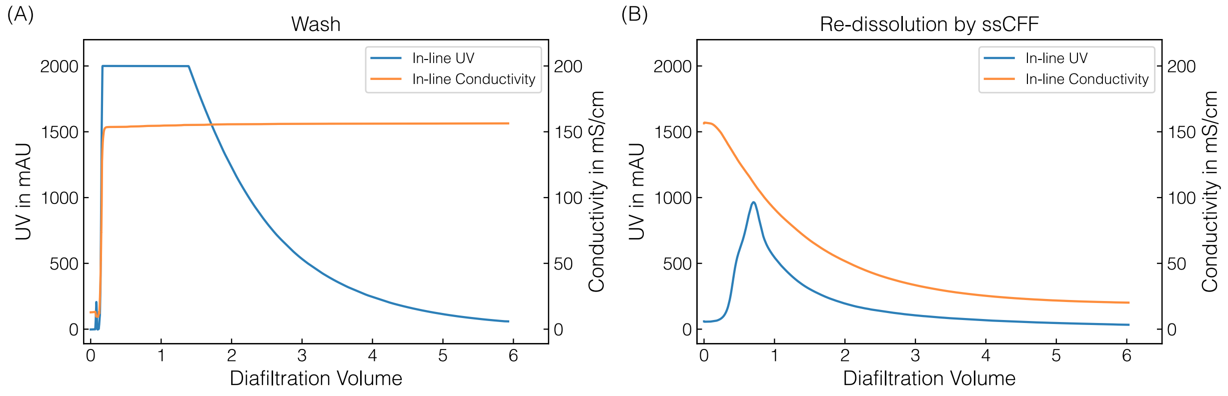

Figure S2. In-line conductivity and UV measurements of wash (A) and re-dissolution (B) by *ssCFF*.
